# Inequalities in Dental Caries Among Primary Schoolchildren in Saudi Arabia: A Cross-Sectional Study

**DOI:** 10.7759/cureus.106801

**Published:** 2026-04-10

**Authors:** Fatimah Alshehri, Elsa Delgado-Angulo, Wael Sabbah

**Affiliations:** 1 Dental Health, College of Applied Medical Sciences, King Saud University, Riyadh, SAU; 2 Dental Public Health, King's College London, London, GBR; 3 Dental Public Health, King’s College London, London, GBR

**Keywords:** children, dental visit, maternal education, primary school student, socioeconomic factors, dental caries

## Abstract

Background: This cross-sectional study aims to investigate the association between socioeconomic and behavioural factors and dental caries among primary schoolchildren in Riyadh, Saudi Arabia.

Methods: A total of 202 first-grade primary school students (mean age 6.2 years; 111 (55%) female) from public schools in Riyadh, Saudi Arabia, participated in the study. Socioeconomic and behavioural data were collected using the World Health Organization (WHO) oral health questionnaire completed by parents, while dental caries, measured using the decayed-missing-filled teeth (dmft) index, was assessed through clinical examination following WHO criteria. Ethical approvals were obtained from King Saud University (Institutional Review Board (IRB) No. 24/1240) and King’s College London (LRS/DP-23/24-40999). Logistic regression was used to assess the association between parents’ education and dental caries, adjusting for age, gender, dental visits in the last year, routine dental visits, oral hygiene, and sugar consumption.

Results: The overall caries prevalence (dmft > 0) was 85.2% (172/202), with a mean dmft score of 5.43 (SE = 0.31). The percentage of children with untreated caries in deciduous teeth was 83.7% (169/202), with a mean of 4.96 (SE = 0.281). In the logistic regression, higher maternal education was negatively associated with caries experience (odds ratio (OR): 0.26, 95% confidence interval (CI): 0.07-0.99). A dental visit within the last year was positively associated with caries experience (OR: 3.46, 95% CI: 1.25-9.64). In contrast, routine dental visits for check-ups were negatively associated with caries experience (OR: 0.27, 95% CI: 0.08-0.90).

Conclusions: Maternal education and consistent routine dental check-ups emerged as significant protective factors against dental caries. Emphasising preventive dental care and parental education may reduce the burden of caries among Saudi children.

## Introduction

Dental caries remains a significant public health concern affecting individuals of all ages and socioeconomic backgrounds. Globally, the World Health Organization (WHO) estimates that over 2 billion individuals are affected by dental caries in their permanent dentition, while approximately 514 million children suffer from caries in their primary teeth [1]. Caries can lead to pain, infection, poor quality of life and difficulties in eating, speaking, and learning [2]. In Saudi Arabia, studies have consistently reported a high prevalence of caries among children, exceeding 70%, with variation across different regions and socioeconomic backgrounds [3-5]. A recent systematic review and meta-analysis conducted in 2024 found that the mean decayed-missing-filled teeth (dmft) index for primary teeth was 4.14, with a prevalence of 75.43% [6]. Despite the decline in these scores compared with previous data, they remain higher than those reported in other countries. Notably, the WHO reports that 53% of Saudi children aged 1-9 years have untreated dental caries, making it one of the highest rates reported globally [7]. In the broader Gulf Cooperation Council (GCC) countries, similarly high caries burden has been documented among schoolchildren, with pooled prevalence reaching over 80% in primary teeth (mean dmft 5.14) [8,9]. Despite the availability of free oral healthcare, established school-based preventive measures such as fluoride application, and ongoing oral health awareness initiatives, inequalities persist in the distribution of dental caries among Saudi children [10]. Numerous studies conducted in Saudi Arabia have consistently identified various factors correlated with increased incidents of dental caries in children, and these factors are directly driving existing oral health inequalities. These include socioeconomic determinants such as parental education levels, geographic location, and household income. Children from lower socioeconomic backgrounds, particularly those in rural areas or with limited parental education, tend to experience a higher rate of dental caries [11,12]. Furthermore, behavioural factors such as dietary habits, oral hygiene practices, and utilisation of dental services also play a significant role. A higher caries incidence is strongly associated with inadequate oral hygiene practices, high sugar intake, and infrequent dental visits [12-14]. The inequalities in dental caries across the population are often exacerbated by the socioeconomic disparities that limit equitable access to oral healthcare, nutritious food, and effective oral health education. Consequently, children from lower-income households and those with less parental education bear a higher burden of dental caries, which enhances the existing oral health inequalities [15]. This highlights the need to examine how the socioeconomic and behavioural factors influence the inequalities in dental caries. Understanding these factors is vital for developing targeted interventions to improve oral health outcomes among the population. While existing research in Saudi Arabia has focused on dental caries prevalence, limited research has explored the inequalities associated with dental caries. Therefore, this cross-sectional study aims to address this research gap by examining the association between key socioeconomic (maternal education level) and behavioural factors (dental visit patterns, oral hygiene practices, and dietary habits) and caries experience (dmft > 0), as well as assessing caries prevalence among primary schoolchildren in Riyadh, Saudi Arabia.

## Materials and methods

Study design and settings

This study employed a cross-sectional design to investigate the prevalence and inequalities in dental caries among first-grade students in primary schools in Riyadh City, Saudi Arabia. From the comprehensive list of all public primary schools in Riyadh obtained from the Ministry of Education, three schools were randomly selected using simple random sampling with computer-generated random numbers. Data collection was conducted during the first semester of the 2024/2025 academic year, specifically between August and December 2024. 

Ethical consideration

Ethical approvals were obtained from the Research Ethics Committees of King Saud University (24/1240/IRB) and King's College London (LRS/DP-23/24-40999). Written informed consent was secured from the children and their parents or legal guardians. A child-friendly version of the consent had been created to explain the nature of the study in an age-appropriate manner. All participants were assured of anonymity and confidentiality, with data assigned to unique identifiers to protect their privacy.

Participants

All eligible first-grade primary school students, aged between 5 and 8 years, within the three selected schools were included, resulting in a final sample of 202 children. Inclusion in the study required students and their parents to provide written informed consent. Students were excluded if consent was declined or if they were medically unfit for oral examination. This sample size was considered adequate for estimating the prevalence of dental caries and examining major socioeconomic and behavioural associations, given the high expected prevalence of dental caries (approximately 75%) reported in recent Saudi meta-analyses [6]. Riyadh City has over 1,200 public primary schools. This study was limited to public schools only, as they serve the majority of Saudi children and allow better control for socioeconomic variability compared with private schools.

Data collection

The data collection process involved a parental questionnaire to assess socioeconomic and oral health-related behaviours, as well as a clinical oral examination for caries assessment. A pre-tested, self-administered questionnaire adapted from the WHO Oral Health Questionnaire for Children, 2013, was used to collect data from parents or guardians (Appendices A-D) [16]. The questionnaire was translated into Arabic, back-translated, and pilot-tested on 15 parents for cultural appropriateness and clarity. Minor wording adjustments were made based on pilot feedback. This instrument gathers information on demographic characteristics (child’s age, gender, location), socioeconomic status (parental education levels), and oral health-related behaviours (dietary habits, oral hygiene practices, dental service utilisation). Once the child and parental informed consent were secured, schools disseminated and collected the questionnaire. The questionnaire took approximately 15 to 20 minutes to complete. Dental caries was assessed through clinical oral examinations conducted by trained and calibrated dentists at the selected schools, using adequate natural light, a plain mouth mirror, a periodontal probe, an explorer, and gauze. The number of decayed, missing, and filled teeth (dmft) was assessed according to the WHO standardised diagnostic criteria. The findings were recorded in the WHO Oral Health Assessment Form for Children, 2013 [16]. Before the study, examiners were trained and calibrated according to the WHO Oral Health Surveys: Basic Methods (2013) [16]. Calibration was performed on 20 children. Inter-examiner reliability for dmft scoring was good (Cohen’s kappa = 0.85). Duplicate examinations were conducted on 10% of the participants during the study to monitor consistency.

Statistical analysis

Data were analysed using Stata Statistical Software, Version 16.0 (StataCorp LLC, College Station, TX). Descriptive statistics summarised participants’ characteristics (frequencies, percentages, means ± SE) and caries status (overall prevalence (dmft > 0), mean dmft scores). Initial bivariate associations between categorical independent variables and caries presence (dmft > 0) were assessed using Pearson's Chi-square tests. To identify factors associated with caries experience, logistic regression analysis was performed. The outcome variable for regression was the presence of caries (dmft ≥ 0), with explanatory variables including demographic, socioeconomic, and oral health-related behavioural factors. Sugar consumption was assessed as a frequency score (0-6) based on the WHO questionnaire items for sugary snacks and drinks per day, with higher scores indicating greater consumption. Results were reported as odds ratios (ORs) with 95% confidence intervals (CIs). Statistical significance was set at *P* < 0.05.

## Results

Participants’ characteristics and caries status

A total of 202 primary schoolchildren participated in the study, yielding a 100% response rate. The sample comprised 111 (55%) females, with a mean age of 6.2 years. The overall caries prevalence (dmft > 0) was 172 (85.2%), with a mean dmft score of 5.43 (SE 0.31) for the primary dentition.

**Figure 1:**
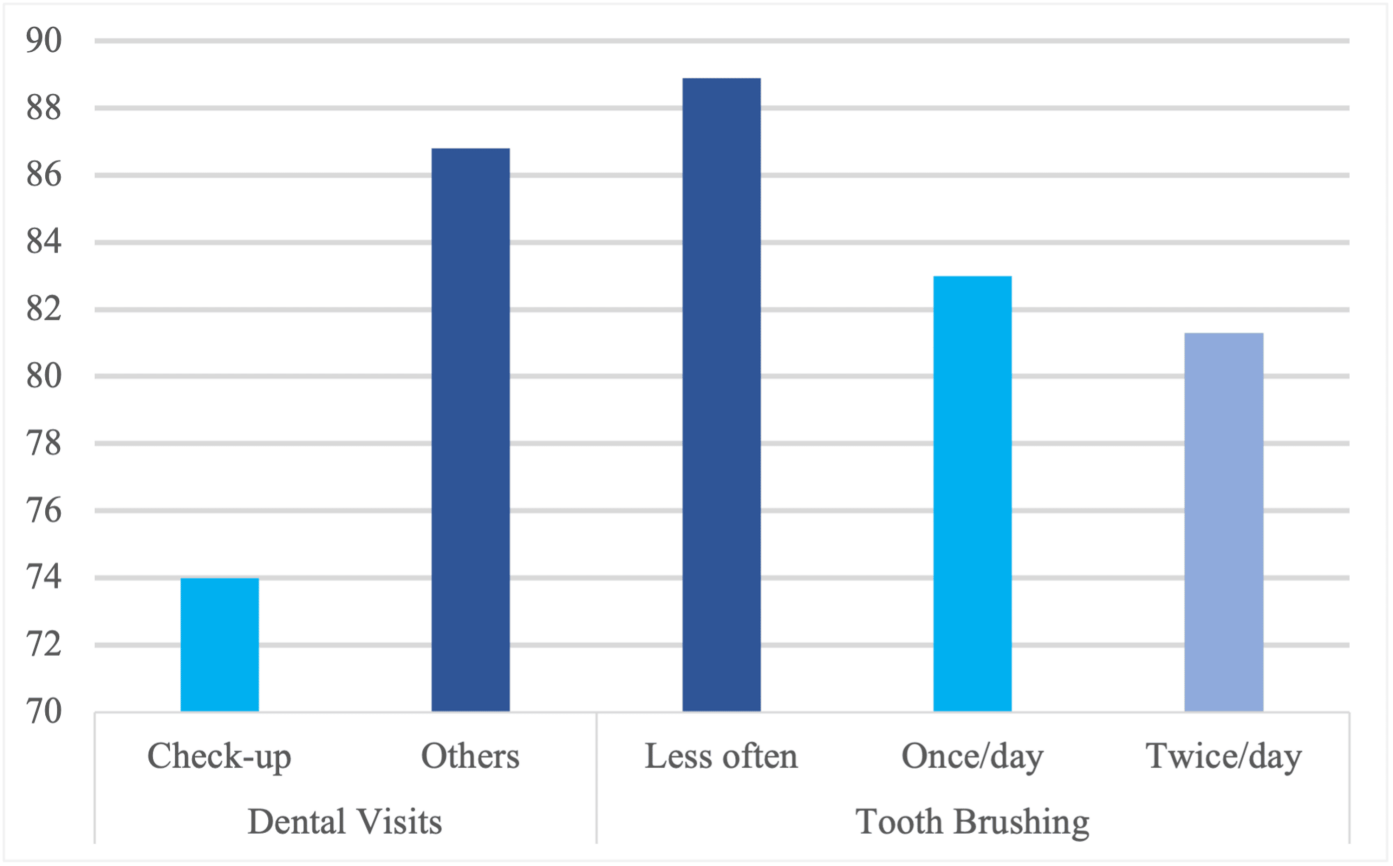
Prevalence of caries experience among five- to eight-year-old children by behaviour.

Variations in caries experience were found to be associated with behavioural factors (Figure 1). A higher caries prevalence was observed among children reporting no prior dental visit or symptom-driven visits (144/166, 86.8%), compared to those who had check-up dental visits (28/36, 77.8%) (Pearson’s χ² (1) = 1.882, *P* = 0.170). Similarly, caries prevalence varied by toothbrushing frequency, with children brushing less than once a day showing the highest prevalence, 88.9% (56/63), followed by those brushing once a day, 84.0% (89/106), and those brushing twice or more a day (27/33, 81.8%) (Pearson’s χ²(2) = 1.104, *P* = 0.576).

**Figure 2:**
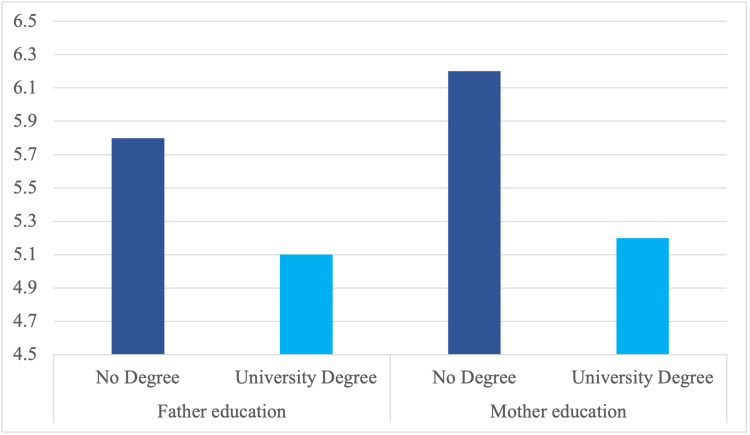
Mean dmft scores among five- to eight-year-old children by parents’ education. dmft, decayed, missing, and filled teeth

Mean dmft scores also varied by parental education (Figure 2). Children whose mother had *No Degree *displayed a higher mean dmft score (approximately 6.2) than those whose mother held a *University Degree* (approximately 5.2), with a similar trend observed for father’s education.

Furthermore, the chi-square test showed a statistically significant association between age group and caries prevalence (*P* = 0.025), indicating that older children aged seven to eight years exhibited a higher caries prevalence (51/54, 94.4%) compared to those aged five to six (121/148, 81.8%) (Pearson’s χ²(1) = 5.036, *P* = 0.025). No other significant associations were found between caries prevalence and sex or other behavioural and socioeconomic factors.

Multivariate logistic regression analysis

Table 1 represents the logistic regression analysis examining factors associated with the presence of dental caries (dmft > 0). Within this model, several factors emerged as independently significant predictors of caries presence. Children aged seven to eight years were significantly more likely to have dental caries (adjusted OR (AOR) = 3.82; 95% CI: 1.06, 13.65; *P* = 0.040) compared to those aged five to six years. Similarly, children who visited a dentist *more than once* were significantly more likely to have caries (AOR = 3.45; 95% CI: 1.27, 9.83; *P* = 0.015) compared to those who visited *less than once/never*. Conversely, children whose last dental visit was for a *check-up* were significantly less likely to have caries (AOR = 0.27; 95% CI: 0.08, 0.90; *P* = 0.034) compared to those who had either a treatment visit or no visit. Additionally, children whose mothers had a *University degree *were significantly less likely to have dental caries (AOR = 0.26; 95% CI: 0.07, 0.98; *P* = 0.047) compared to those whose mothers had *less than a university* education.

**Table 1:** Logistic regression analysis showing adjusted odds ratios (AORs) for factors associated with the presence of dental caries (dmft ≥ 0) among primary schoolchildren in Riyadh (N = 202). *P < 0.05. 95% CI, 95% confidence interval; dmft, decayed, missing, and filled teeth

Characteristics	P-value	AOR	95% CI
Sex			
Male (Reference)	-	-	-
Female	0.079	0.45	0.18-1.09
Age			
5-6 years (Reference)	-	-	-
7-8 years	0.040*	3.82	1.06-13.65
Visited a dentist last year			
Less than once/never (Reference)	-	-	-
More than once	0.015*	3.45	1.27-9.83
Reason for last dental visit			
Treatment/No visit (Reference)	-	-	-
Check-up	0.034*	0.27	0.08-0.90
Tooth brushing			
Less than once a day (Reference)	-	-	-
Once a day	0.189	0.49	0.17-1.14
Twice or more	0.332	0.53	0.15-1.91
Greater sugar consumption (per unit increase in score)	0.968	0.99	0.86-1.16
Father's education			
Less than university (Reference)	-	-	-
University degree	0.051	3.18	0.99-10.17
Mother's education			
Less than university (Reference)	-	-	-
University degree	0.047*	0.26	0.07-0.98

## Discussion

This study aimed to assess the prevalence of dental caries and identify the associated socioeconomic and behavioural factors among first-grade primary schoolchildren in Riyadh, Saudi Arabia. The observed high caries prevalence of 172 (85.2%) and a mean dmft score of 5.43 (SE: 0.31) for primary dentition aligns with previous systematic reviews and meta-analyses reporting high rates often above 70% among Saudi children [3-6]. These findings underscore a serious and persistent public health challenge in Saudi Arabia. Similar high caries burden has been documented across the GCC countries, with pooled prevalence estimates of approximately 65% in permanent teeth and over 80% in primary teeth [8,9]. This indicates that the substantial caries burden observed in the present study aligns with the broader regional pattern, despite economic development and the availability of free oral healthcare services in the Gulf region.

The multivariate logistic regression analysis revealed several predictors of caries presence. Age emerged as a significant factor, with older children aged from seven to eight years showing nearly four times the likelihood of having caries compared to the younger children aged five to six years. This finding is in line with the fact that dental caries is a cumulative disease that develops gradually, with longer exposure to a cariogenic environment and other risk factors leading to greater accumulation of lesions over time [17].

Higher maternal education and routine dental check-ups were significantly associated with lower caries experience. Educated mothers often possess greater health literacy, enabling them to better understand and implement effective oral hygiene practices, make healthier dietary choices for their families, and more consistently prioritise and access preventive health services for their children [11,12]. They frequently serve as primary educators and role models for healthy habits within the household, directly influencing a child's early health behaviours. Similarly, visits for routine check-ups significantly reduced the odds of caries by 73% compared to symptom-driven visits or no visits. This emphasises the critical role of proactive, preventive dental care in early detection and intervention, as opposed to reactive treatment. Despite the availability of free dental care in Saudi Arabia, dental visits are only symptomatic when experiencing symptoms or in emergencies [18-20]. Afeef et al. further highlighted this by reporting that most Saudi children's initial dental visits occur late, between the ages of 3 and 10 years, primarily when they experience pain or cavities [21]. This irregular utilisation of dental health services contributes to the high prevalence of untreated dental caries, which affirms its position as the primary oral health concern among Saudi children.

Conversely, children who reported visiting a dentist *more than once* in the past 12 months were significantly more likely to have caries. This result could be interpreted as children with existing or more severe caries are probably those who require and receive more frequent dental attention for treatment, rather than visits for a primary preventive function [12]. This highlights the distinction between symptom-driven reactive care, which often indicates existing disease and the consistent routine preventive check-ups.

Regarding other factors, the multivariate model did not find statistically significant associations with caries presence for sex, tooth brushing frequency, father's education, or sugar consumption. This absence of significance, particularly for toothbrushing frequency and sugar consumption, contrasts with extensive evidence consistently highlighting their critical role in caries aetiology [14,17,22]. This difference might be due to the specific age group, parent-reported data limitations or confounding factors. For brushing frequency, descriptive trends (Figure 1) suggested variation, but multivariate analysis, along with counter-intuitive prevalence patterns, suggests potential reverse causality. Regarding sugar consumption, its non-significant association does not imply a lack of biological effect. The consumption of cariogenic sugars is uniformly high across the study population, acting as a widespread baseline risk factor. Thus, a lack of variation in sugar consumption within the sample limits its ability to emerge as a significant predictor, despite the overall high caries prevalence, underscoring its pervasive impact. Furthermore, the non-significant finding for father's education contrasts with the protective effect of maternal education and warrants further investigation into specific cultural or socio-economic dynamics in this population that might differentiate the roles of maternal and paternal education.

This study offers valuable insights into dental caries inequalities among a vulnerable age group of primary schoolchildren in Riyadh City. Key strengths include the use of standardised WHO methods for data collection and caries assessment, which enhanced comparability with other populations, and the multi-staged sampling approach to ensure representativeness within this urban setting.

This study has some limitations. The cross-sectional design restricts causal inference. Additionally, reliance on parent-reported information introduces potential recall or social desirability bias. The sample was restricted to public schools in Riyadh (*N* = 202), which may limit generalisability and introduce selection bias due to differences in socioeconomic status and dental care access compared with private schools. Furthermore, the positive association observed with recent dental visits likely reflects reverse causality, as children with existing caries are more likely to seek treatment. Future research with larger, more geographically diverse samples across Saudi Arabia is necessary to improve generalisability and uncover additional associations.

## Conclusions

This study addressed the prevalent issue of dental caries and associated inequalities among Saudi primary schoolchildren. The main findings confirm an alarmingly high caries prevalence, with higher maternal education and routine dental check-ups being significantly associated with lower odds of dental caries. These results highlight the urgent need for public health initiatives to promote maternal oral health literacy and improve the accessibility and utilisation of regular dental care. Such targeted interventions are crucial for effectively mitigating the burden of dental caries and reducing persistent oral health inequalities among children in Saudi Arabia.

## Figures and Tables

**Figure 3:**
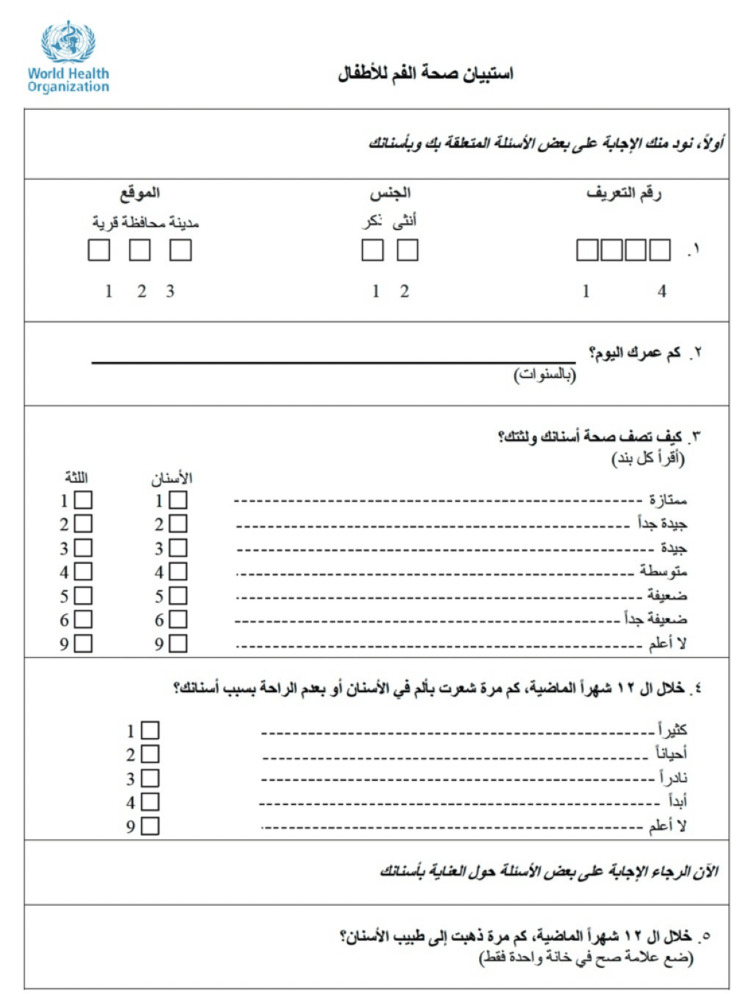
WHO Oral Health Questionnaire for Children (Page 1). Source: [13].

**Figure 4:**
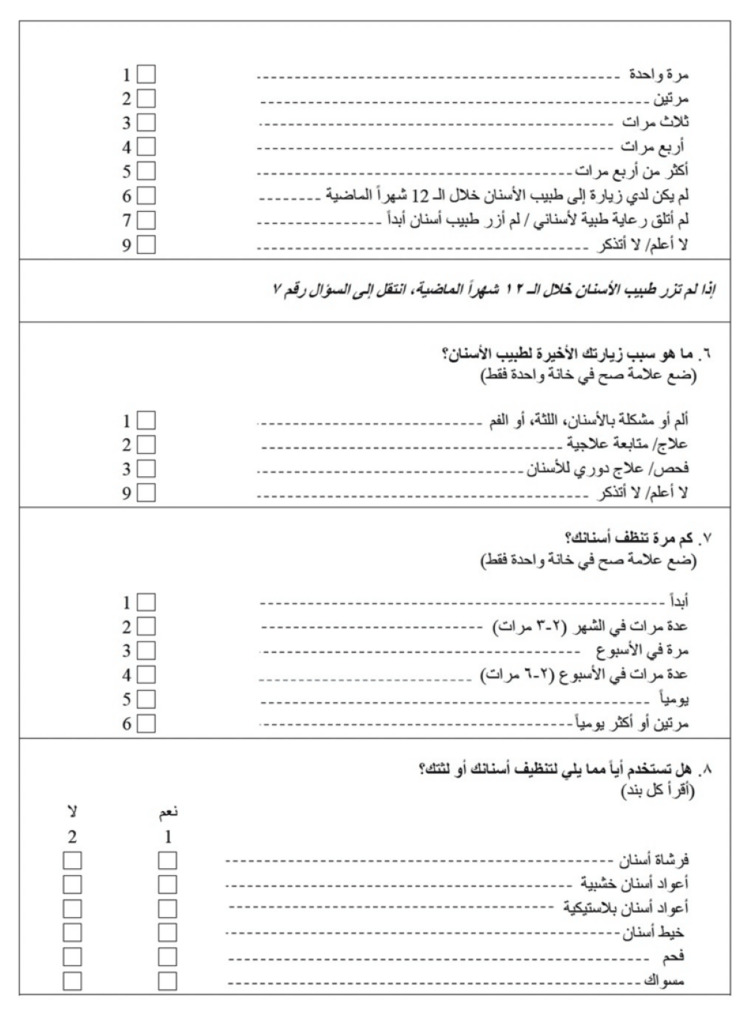
WHO Oral Health Questionnaire for Children (Page 2) Source: [13].

**Figure 5:**
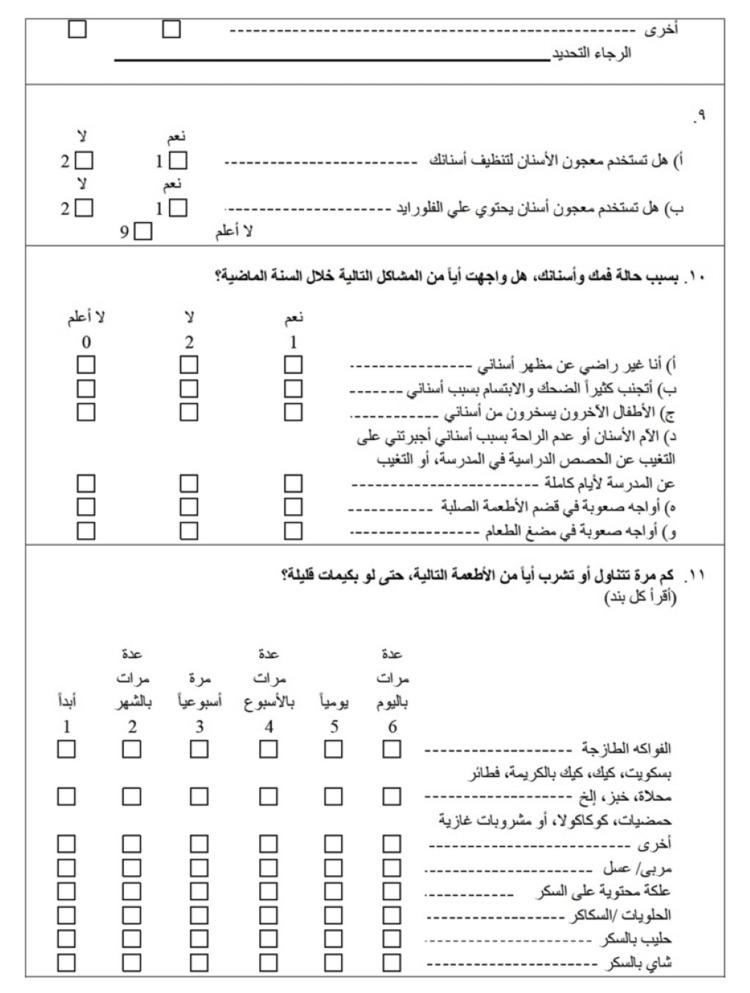
WHO Oral Health Questionnaire for Children (Page 3). Source: [13].

**Figure 6:**
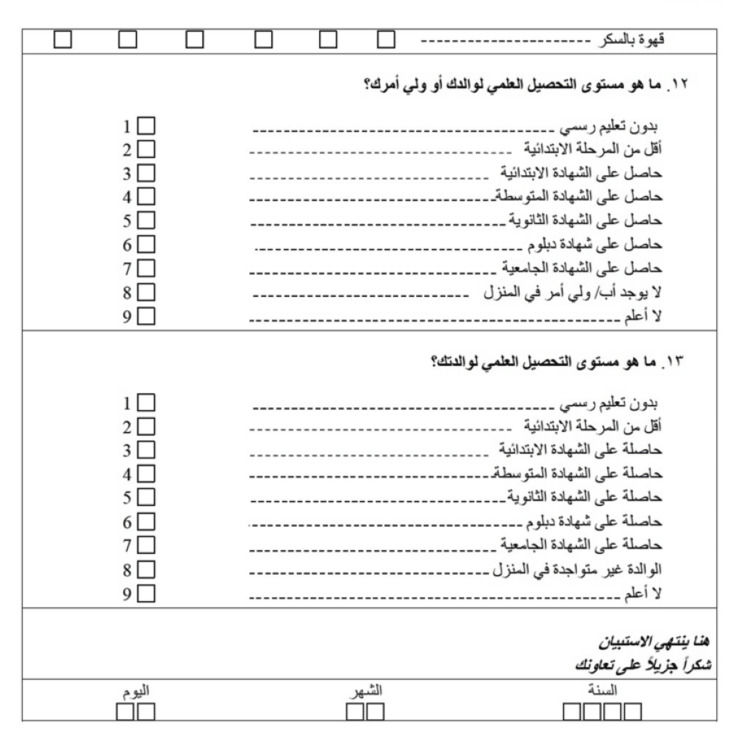
WHO Oral Health Questionnaire for Children (Page 4). Source: [13].

## Appendices

Appendix A 

Appendix B 

Appendix C 

Appendix D 
